# Comparison of non-thermally extracted polysaccharides from fresh wolfberry with conventionally extracted polysaccharides from dried wolfberry

**DOI:** 10.1371/journal.pone.0355472

**Published:** 2026-08-11

**Authors:** Ru Zhang, Zhuan Nan, Caifang Wang, Raorao Li, Yanping Li, Jianbao Ding, Hao Wang, Zhexiong Yu, Jin Yang

**Affiliations:** 1 School of Chemistry and Chemical Engineering, North Minzu University, Yinchuan, Ningxia, China; 2 School of Pharmaceutical Sciences, Capital Medical University, Beijing, China; 3 Institute of Chinese Materia Medica, China Academy of Chinese Medical Sciences, Beijing, China; 4 Ningxia Wuxing Science and Technology Co., Ltd., Yinchuan, Ningxia, China; 5 School of Preparatory Education, North Minzu University, Yinchuan, Ningxia, China; 6 Ningxia Tianren Goji Biotechnology Co., Ltd., Zhongning, Ningxia, China; 7 Key Laboratory for Chemical Engineering and Technology, State Ethnic Affairs Commission (North Minzu University), Yinchuan, Ningxia, China; Konkuk University, KOREA, REPUBLIC OF

## Abstract

*Lycium barbarum* polysaccharides (LBPs) are important bioactive constituents of wolfberry, but polysaccharides prepared directly from fresh wolfberry under non-thermal conditions remain insufficiently characterized. This study developed a non-thermal extraction strategy to obtain polysaccharides from fresh wolfberry (FLBPs) and compared them with polysaccharides conventionally extracted from dried wolfberry (DLBPs). By adjusting the relative density of pulp to 1.06 and applying an 85% alcohol precipitation, FLBPs were successfully prepared without heating. FLBPs and DLBPs showed comparable total carbohydrate and protein contents, whereas DLBPs contained significantly more bound phenolics (*p* < 0.001). The two crude polysaccharides differed in molecular-weight distribution, monosaccharide composition, morphology, Congo red response, particle size, zeta potential, apparent viscosity, and cellular bioactivities. Compared with DLBPs, FLBPs displayed lower bound phenolic content, a narrower molecular weight distribution, stronger cytoprotective activity against H_2_O_2_-induced oxidative stress in HepG2 cells, and less induction of NO release in RAW264.7 macrophages. These findings indicate that non-thermally extracted FLBPs can serve as a fresh-material reference for evaluating preparation-associated differences in wolfberry polysaccharides and provide clues for future studies on the roles of drying, extraction, and associated components in shaping the quality of LBPs.

## Introduction

Wolfberry, the fruit of *Lycium barbarum* L., is one of the most popular herbal medicines and functional food ingredients in tonic food [1]. *Lycium barbarum* polysaccharides (LBPs) are known as the most important active components in the herbal medicine and have attracted increasing attention for their significant biological activities, including antioxidation, anti-inflammation, immune regulation, and liver protection, and potential medicinal value [2,3]. Their content and structure directly influence the nutritional value and health benefits [4].

Traditionally, fresh berries are prone to spoilage due to their high moisture content and therefore need to be dried to extend the shelf life of wolfberry and facilitate transportation and storage [5]. Most drying methods involve heating and rapid dehydration, which can trigger a series of complex physicochemical changes that inevitably affect their active components [6]. Previous studies have shown that drying methods affect the quality of wolfberry and can alter the physicochemical properties of plant polysaccharides [7,8]. These changes may involve molecular-weight redistribution, aggregation, hydrogen-bond rearrangement, and processing-related chemical reactions [9,10]. However, most studies have evaluated polysaccharides obtained from dried materials. Such designs are useful for ranking drying methods, but they provide limited information about how these preparations differ from a fresh-material, minimally thermally perturbed reference.

Therefore, this study aimed to develop a non-thermal extraction strategy for obtaining polysaccharides from fresh wolfberry (FLBPs) and to compare the obtained FLBPs with polysaccharides conventionally extracted from dried wolfberry (DLBPs). The comparison was intended to provide a fresh-material reference for evaluating differences in chemical composition, molecular-weight distribution, monosaccharide profile, morphology, conformation-related behavior, solution properties, and cellular bioactivities between the two polysaccharides preparations. By integrating these compositional, macromolecular, colloidal, and cellular descriptors, this study sought to identify the shared and divergent features of the two polysaccharide preparations and to provide a reference dataset for subsequent process-oriented studies of LBPs.

## Materials and methods

### Reagents and materials

The fresh wolfberry used in the experiment was provided by Ningxia Tianren Goji Biotechnology Co. Ltd and identified as the mature fruit of *Lycium barbarum* L. (Solanaceae) by Professor Jianbao Ding of Ningxia Wuxing Technology Co. Ltd. The fresh berry was dried using hot air-drying method according to the previous report [7]. Briefly, the fresh wolfberries were surface-dewaxed by washing with aqueous sodium carbonate solution, followed by thorough water rinsing and draining. The cleaned samples were then subjected a constant drying at 60 ℃ until the moisture content was less than 13%. Fresh wolfberries were processed for polysaccharide preparation as soon as possible after receipt. A portion of the material was stored at −20 °C as retained samples and for short-term preservation before analysis. Repeated freeze-thaw cycles were avoided.

Monosaccharide standards, including rhamnose (Rha), arabinose (Ara), galactose (Gal), glucose (Glc), galacturonic acid (GalA), Mannose (Man), Xylose (Xyl), Glucosamine (GlcN), and glucuronic acid (GlcA), were purchased from Merck Ltd. (Shanghai, China). Coomassie Brilliant Blue G250, bovine serum albumin, gallic acid, Folin’s reagent, 2,4,6-tris(2-pyridyl)-1,3,5-triazine (TPTZ), ferric chloride hexahydrate, 2,2’-azino-bis(3-ethylbenzothiazoline-6-sulfonic acid) diammonium salt (ABTS), and Congo red were obtained from Shanghai Titan Technology Co., Ltd. (Shanghai, China). All other reagents were of analytical grade.

Cell line HepG2 and Cell Counting Kit-8 (CCK-8) were purchased from Wuhan Servicebio Technology Co. Ltd. (Wuhan, China). The RAW264.7 cell line and the complete culture medium (CCM) were provided by Wuhan Pricella Biotechnology Co. Ltd (Wuhan, China). Lipopolysaccharide (LPS) was provided from Merck Ltd. (Shanghai, China). The IL-6 ELISA kit was purchased from Beijing Puliailai Gene Technology Company (Beijing China).

### Extraction of FLBPs

The preparation processes of FLBPs were developed guided by *in vitro* antioxidant activities, namely, ferric ion reducing power (FRAP) and ABTS·^+^ radical scavenging [11]. The fresh wolfberry was crushed with a colloid mill (JM-L50, Wenzhou Qiangzhong Machinery Technology Co. Ltd, Wenzhou, China). The relative density of pulp was measured in accordance with the method specified in the *Chinese Pharmacopoeia* (2020 Edition, ChP2020) [12]. The pulp was adjusted for the appropriate density with pure water and then subjected to ethanol precipitation. The mixture was kept overnight. The precipitate (FLBPs) was collected using centrifugation (4500 × g, 15 min, HDC-18K Plus High-speed Centrifuge, Shanghai Titan Scientific Co. Ltd, Shanghai, China) and dialyzed using ultrafiltration membranes (Beijing BOAOtoda Technology, Co. Ltd., Beijing, China) with the MW cutoff (MWCO) of 3 kDa. The retained liquid was collected and lyophilized (FD-1C-50, Beijing Biocool Co. Ltd, Beijing, China). The percentage of FLBPs yield (%) was calculated as follow:

FLBPs yield (%) = (weight of dried FLBPs/weight of total solid of pulp) × 100%

here, weight of total solid of pulp was determined according to the method recorded in ChP2020 [12].

Pulp’s density and the gradient alcohol precipitation are the key parameters affecting the efficiency of alcohol precipitation process [13,14]. Consequently, these two variables were selected for the single-factor tests in an antioxidant activities-oriented manner. The pulp’s density was set 1.02, 1.03, 1.04 and 1.06, respectively, and the concentration for ethanol precipitation was set from 50% to 90%. During the optimization of experimental variables, just one factor in each experiment was changed, while the remaining factors remained unchanged. The products during the process optimization were dissolved in pure water, and then its ferric ion reducing power (FRAP) and ABTS·^+^ radical scavenging capacity were tested.

### Preparation of DLBPs

DLBPs were extracted according to the patent method [15] with some modifications. Briefly, the dried wolfberries were decocted twice with 8 times pure water for 2 h each time. The decoction was rotary evaporated to an appropriate volume. Then, 95% alcohol (v/v) was added until the final ethanol concentration reached 85%. The mixture was maintained overnight. The precipitate was collected by centrifugation (4500 × g, 15 min) and dialyzed using ultrafiltration membranes with MWCO of 3 kDa. The retained liquid was collected and lyophilized, labeled as DLBPs.

### Chemical characterizations of FLBPs and DLBPs

Using glucose as the standard, the contents of total carbohydrate (TCC) of FLBPs and DLBPs was determined by phenol-sulfuric acid method [16]. The total phenolics contents (TPhC) and total protein contents (TPrC) were measured by means of Folin Ciocalteu method [17] and Bradford method [12], respectively.

The monosaccharides composition was measured by pre-column derivation coupled with HPLC method as previously described by Liu et al [18]. 5 mg polysaccharide samples were hydrolyzed by 2 M trifluoroacetic acid at 110 ℃. The hydrolysates and standard monosaccharides, including Ara, Gal, Glc, Rha, Man, Xyl, GlcN, GalA and GlcA were dissolved in 100 µL NaOH solution (0.3 M), and added 100 µL 1-phenyl-3-methyl-5-pyrazolone (PMP) methanol solution. The reactive solutions were carried out at 70 ℃ for 1 h, and then cooled to room temperature. The reaction products were neutralized with 0.3 M HCl and extracted repeatedly by 1 mL chloroform. The aqueous phases were filtered through 0.45 µm filter for HPLC analysis. The HPLC analysis was performed on a Thermo U3000 HPLC system. The analytic samples were injected into a ZORABAX Eclipse XDB-C18 column (4.6 × 250 mm, 5 µm, Agilent Technologies, USA) and analyzed under 30 ℃. The column was eluted with phosphate buffer (pH 6.8) and acetonitrile (83:17) at the flow rate of 0.8 mL/min. The detection wavelength was 250 nm and chromatographic peaks were identified by comparing their retention time with those of the standards.

According to the previously described [19], the molecular weight (MW) distributions of FLBPs and DLBPs were measured using a Waters high performance gel permeation chromatography (HPGPC) system, equipped with a Waters 1525 liquid chromatograph, 2414 Refractive Index Detector (RI). The samples were dissolved in NaCl solution and injected for analysis. Two tandem columns, namely, OHpak SB-804 HQ and SB-806M HQ2 (300 × 8 mm, Shodex Co., Ltd, Tokyo, Japan) were chosen for separating the samples and eluted with 0.05 mol/L aqueous solution of NaCl at the flow rate of 0.5 mL/min at temperature of 40 ℃. Pullulan polysaccharides with different MW were used to establish a standard curve. The Empower3 software was utilized to process MW data.

The attenuated total reflectance (ATR) infrared spectrum of FLBPs and DLBPs were recorded by Nicolet is50 Fourier transform infrared spectrometer (Thermo Fisher Scientific Instrument Co., Ltd., USA) in the range of 4000–400 cm^-1^ with a resolution of 4 cm^-1^. The spectral data were scanned 32 times and collected by OMNIC 9.

The triple helix structure of the test samples was studied by Congo red experiment [20]. 2 mg/mL polysaccharide solution was combined with 160 μg/mL Congo red solution and NaOH solutions with different concentrations (0, 0.05, 0.1, 0.15, 0.2, 0.4, 0.8, 1.2, 1.6, and 2.0 mol/L) in a brown volumetric flask, respectively. Using distilled water as the blank group, the absorption wavelength of the Congo red in varying concentrations of the NaOH solution was measured with an ultraviolet spectrophotometer (TU-1901, Beijing General Analytical Instrument Co., Ltd., Beijing, China) scanning during 400–600 nm.

### The morphology analysis of FLBPs and DLBPs

A scanning electron microscope (FEI Tecnai 220, JEOL Ltd., Japan) was employed to observe the surface morphology of the FLBPs and DLBPs. A small amount of each of sample was mounted onto a copper metal subs, followed by gold sputter-coated to enhance conductivity. The microstructural features were then examined and imaged at a magnification of 200× and 500× under an accelerating voltage of 5 kV, respectively.

The particle size distribution data and zeta potential values of polysaccharides samples were measured with a Zetasizer Nano ZS90 (Malvern Instruments, UK) with each sample being analyzed in triplicate.

### The viscosity of FLBPs and DLBPs

The apparent viscosities of FLBPs and DLBPs solutions (0.2 g/mL) were measured over a shear rate range of 0.1–100 s^-1^ at 25 °C using an MCR 302 rheometer (Anton Paar, Austria) [21].

### Antioxidant activity of samples

#### Ferric ion reducing antioxidant power (FRAP) for optimizing the preparation of FLBPs.

The ferric reducing antioxidant power were determined according to the method of literature [22]. 0.20 mL of different concentrations of polysaccharides solutions were mixed with 3.90 mL fresh FRAP working solution, respectively, and incubated for 10 min at 37 ℃. Using deionized water and Vc as the blank control and positive control, respectively, the absorbance of the mixture was measured at 593 nm using a Varioskan LUX microplate reader (Thermo Fisher Scientific, CA, USA). The FRAP values were reported as mg of Vc equivalents per g of dry weight (DW).

#### ABTS·^+^ radical scavenging capacity for optimizing the preparation of FLBPs.

The ABTS·^+^ radical scavenging assay of samples was measured according to the previously described [23]. 0.20 mL of different concentrations of the samples were mixed with 3.90 mL freshly prepared ABTS·^+^ working solution. Under lightproof conditions, the reaction was carried out at room temperature for 6 min. Using deionized water and L-ascorbic acid as the negative and positive control, respectively, the absorbance of the reaction solution was measured at 734 nm using a microplate reader. The capacity of scavenging ABTS·^+^ radical was calculated with the following equation:

Scavenging activity (%) = [1-(A_1_-A_2_)/A_0_] × 100%

where, A_0_, A_1_ and A_2_ are the absorbance values at 734 nm of the negative control, the sample solution, and sample solution without ABTS·^+^ radical.

#### Protective effects of FLBPs and DLBPs against hydrogen peroxide (H_2_O_2_) – induced oxidative stress in HepG2 cells.

The HepG2 cells were incubated in 96-well plates (1 × 10^5^ /mL) at 37 ℃ under a humidified atmosphere condition containing 5% CO_2_ for 24 h. The culture medium was replaced with 100 µL of fresh DMEM or DMEM containing various concentrations of H_2_O_2_ solution (50, 100, 200, 400, 600, 800 and 1000 µM) at 37 ℃ under the same atmosphere condition for 12 h after incubation, respectively. Replacing the culture medium by 100 µL of fresh DMEM, 10 µL CCK-8 solution was added to treat cells for 1 h. Then, the absorbance was read at 450 nm using a microplate reader.

The culture medium of incubated cells was replaced with 100 µL of fresh DMEM or DMEM containing various concentrations of polysaccharides solution (25, 50, 100, 200, and 400 µg/mL) at 37 ℃ under a humidified atmosphere condition containing 5% CO_2_ for 24 h, respectively. After incubation, the treated cells were processed with CCK-8 using the same method. The minimum toxic concentration (MTC) was determined by measuring the absorbances at 450 nm.

HepG2 cells were treated with 100 µL different concentrations of polysaccharides solution (50, 100, and 200 µg/mL) and incubated in DMEM, respectively. Then, the cells were incubated with DMEM including H_2_O_2_ solution (600 µM) for 12 h. The viability of the cells was determined by the CCK-8 assay and the protective effect of polysaccharides on H_2_O_2_-reducing oxidative stress in HepG2 cells were determined with the viability of cells by the CCK-8 assay [24].

#### Effects of FLBPs and DLBPs on NO and IL-6 release from RAW264.7 cells.

Mouse macrophage RAW264.7 cells were cultured during DMEM high-glucose media supplemented with 10% FBS and 1% penicillin/streptomycin at 37 ℃ under 5% CO_2_ conditions. 2.5 × 10^5^ cells were seeded in 24-well plates and incubated for 24 h. subsequently, the media was removed. Then, the experimental group was added to the medium containing 0.1 mg/mL polysaccharides, with a positive control group (1 μg/mL LPS) and a negative control group set up simultaneously. The 24-well plates were again incubated at 37 ℃ for 24 h. After drug intervention, the supernatant of culture medium was aspirated and centrifuged at 3000 rpm for 10 min to obtain the tested solution for further analysis. Each sample was performed with 3 biological replicates.

For determination of NO release, the Griess method was used [25]. Specifically, the tested samples were transferred to 96-well plates and sequentially 50 µL, subsequently 50 µL Griess A and 50 µL Griess B added into each well. The plate was kept at 37℃ for 15 min. The absorbance of reaction solution was recorded at 540 nm using a microplate reader. The content of NO was calculated using the standard curve established with serial concentrations of NaNO₂ by the Griess method.

For determination of IL-6, according to the instructions of the ELISA kit, specifically, either (100 μL of the prepared series of IL-6 standard solutions) or (25 μL of each sample solution and 75 μL of assay buffer) were added into the 96-well plate. All other operations were performed strictly in accordance with the kit’s instructions. Finally, measurements were taken at 540 nm and 570 nm using a microplate reader to generate the IL-6 standard curve. Based on the standard curve and the OD values of each sample (OD_450nm_ - OD_570nm_), the IL-6 concentrations in the test samples were calculated.

### Statistical analysis

Data are presented as the mean ± standard deviation (SD) of three independent measurements. Statistical significance was evaluated using T-tests or analysis of variance (ANOVA) combined with Dunnett’s multiple range test, and *p* < 0.05 was defined as statistically significant.

## Results and discussion

### Optimization of the polysaccharides’ extraction process from fresh wolfberry

Alcohol precipitation is commonly used for polysaccharide purification and a process can also be employed in the industrial field [26]. Generally, the relative density of solution and ethanol concentration may affect the ethanol precipitation process [27]. An increase in relative density raises the frequency of molecular interaction which is conducive to precipitate formation [28]. With the addition of alcohol, polysaccharides with different physicochemical properties are gradually precipitated and separated from the small molecule compounds or oligosaccharides [29]. Therefore, these two variables were selected for the single-factor test to optimize the polysaccharides extraction process from fresh wolfberry.

**Fig 1:**
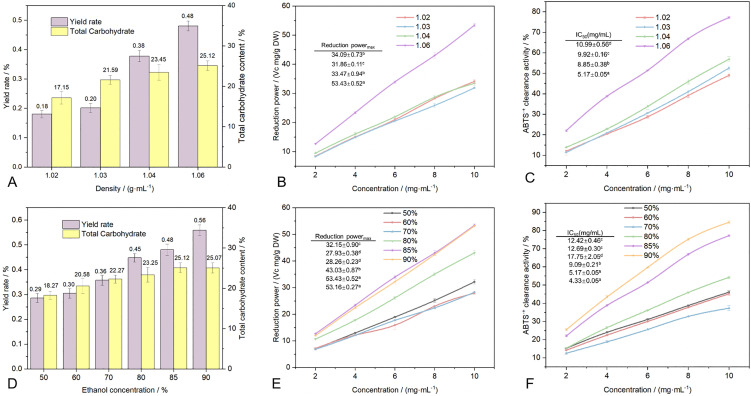
Effects of the relative density of pulp and the concentration of alcohol precipitation on the yield rate of precipitation, total carbohydrate content, the ferric ion reducing antioxidant power (FRAP), and ABTS·^+^ radical scavenging capacity, respectively. (A, D) the influences of the relative density and the concentration of alcohol precipitation on the yield of precipitation and the total carbohydrate content, respectively; (B, E) the effects of different relative densities and concentration of alcohol on the FRAP, respectively; (C, F) the effects of different relative densities and ethanol concentrations on ABTS**·**^**+**^ radical scavenging capacity, respectively. Values are means of triplicate determination ± standard deviations with different letters are significantly different at *p* < 0.05.

Under fixed conditions (ethanol concentration of 85%), the effect of the relative densities of pulp on the extraction rate of polysaccharides were tested. As shown in Fig 1A, the precipitation yields and the polysaccharide contents increased rapidly with the increasing in relative density from 1.02 to 1.06, reaching 0.48% and 25.12%, respectively. This observation suggested that the number of polysaccharide molecules per unit volume rose as the relative density of solution increased, which increased the probability of intermolecular forces forming. Thereby, it became easier for large-particle aggregates to form and precipitate. The ferric ion reducing powers of samples obtained under different densities showed a dose-effect relationship (Fig 1B). Within the test range, the maximum FRAP value of the sample obtained at a relative density of 1.06 was 53.43 ± 0.52 Vc mg/g DW, which was significantly higher than that of other samples (*p* < 0.05). The ABTS·^+^ radical scavenging capacity of the samples also exhibited the same trend (Fig 1C), and the IC_50_ value of the precipitate obtained at the maximum relative density was 5.17 ± 0.05 mg/mL, which was significantly lower than that of other samples (*p* < 0.05). This trend was also positively correlated with the polysaccharide content in the samples. The phenomenon indicated that high density was conducive to the enrichment of antioxidant substances. Therefore, the relative density of pulp at 1.06 was selected for subsequent experiments.

Under constant conditions (with the relative density of pulp at 1.06), the effect of alcohol concentration on the alcohol precipitation process was studied. As shown in Fig 1D, the precipitation yields and polysaccharides contents gradually increased as the ethanol concentration raise from 50% to 90%, reaching 0.56% and 25.07%, respectively. Polysaccharides substituted with hydrophobic groups tend to precipitate at lower ethanol concentration, while those with strong hydrophilicity need higher concentration for precipitation [30]. Thus, higher precipitation yield and polysaccharides content can be expected obtaining from higher alcohol concentration. Numerous studies suggested that natural polysaccharides would be the potential antioxidants [31]. A higher polysaccharides content indicted stronger antioxidant activity. When the alcohol precipitation concentration reached 90%, the FRAP value and IC_50_ value of ABTS·^+^ radical scavenging of samples were 53.16 ± 0.27 Vc mg/g DW and 4.33 ± 0.05 mg/mL, respectively, which exhibited significant differences from all samples except the one obtained with 85% alcohol precipitation (Fig 1E and 1F). Comparison of antioxidant capacities of 85% and 90% alcohol precipitated products suggested there were no significant difference between them. Considering technical and economic factors, an ethanol concentration of 85% was more advantageous.

The single-factor tests analysis provided the optimal conditions for preparation polysaccharides from fresh wolfberry (FLBPs): the relative density at 1.06 and alcohol precipitation concentration of 85%. Notably, the FLBPs was obtained under non-heating conditions during the preparation process according to the optimum terms.

### Chemical properties of FLBPs and DLBPs

The chemical components of FLBPs and DLBPs are listed in Table 1. As shown in Table 1, FLBPs contained more saccharides, while DLBPs comprehended higher content of protein. However, there were no significant difference in these two components between FLBPs and DLBPs. Interestingly, DLBPs contained significantly higher total phenols than that of FLBPs (*p* < 0.001). The observation indicated the possibility of drying and preparation-associated aggregation between phenolics and saccharides or protein [9].

**Table 1:** Analysis of the main component contents and monosaccharide composition of FLBPs and DLBPs.

Index		FLBPs	DLBPs
**TCC (%)**		65.36 ± 1.34	65.06 ± 5.07
**TPrC (%)**		6.99 ± 0.24	8.73 ± 0.34
**TPhC (%)**		3.01 ± 0.01^***^	4.12 ± 0.02
**Monosaccharide composition (mol%)**	Man	3.78	3.87
	GlcN	2.67	2.20
	Rha	2.87	3.61
	GlcA	2.71	2.24
	GalA	3.72	2.78
	Glc	54.01	37.18
	Gal	12.91	13.96
	Xyl	0.56	1.72
	Ara	16.77	32.44

TCC, TPrC, and TPhC values are expressed as mean ± SD (n = 3). Monosaccharide composition is expressed as relative molar percentage. ***p < 0.001 compared with DLBPs.

**Fig 2:**
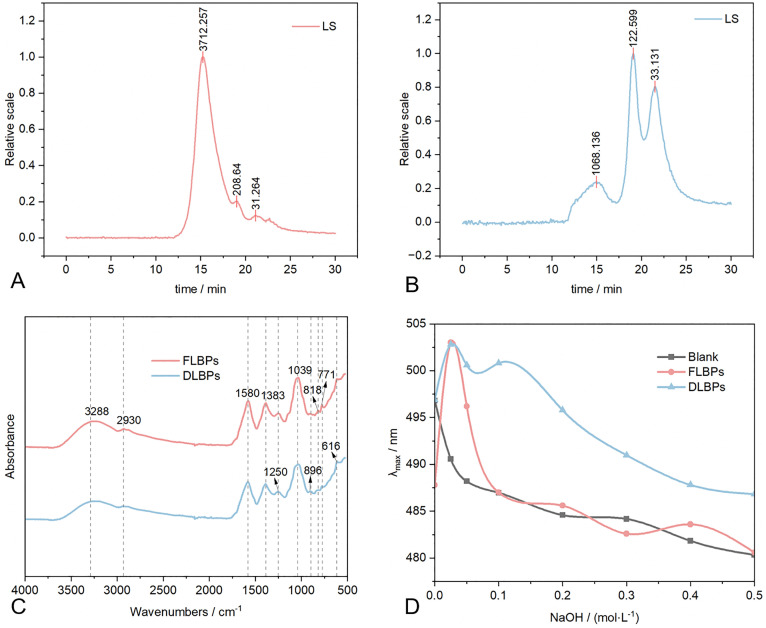
The chemical characteristics of FLBPs and DLBPs. (A, B) HP-GPC chromatogram of FLBPs and DLBPs, respectively. The numbers on each peak represent the weight-average molecular weight (MW) values. (C) Fourier transform infrared spectra of FLBPs and DLBPs. (D) the maximum absorption wavelengths (λmax) of Congo red (blank), Congo red + FLBPs, Congo red + DLBPs at different concentrations of sodium hydroxide solutions, respectively.

Monosaccharides are the basic units that constitute polysaccharides and determine their specific structure and biological activity [32]. The pre-column derivation coupled with HPLC method measured the monosaccharide and uronic acid composition of FLBPs and DLBPs, together with the calculated molar percentages, were displayed in Table 1 and S1 Fig. The two crude polysaccharides contained the same major monosaccharide types, namely, Ara, Xyl, Gal, Glc, GlcA, GalA, Rha, GlcN and Man, but differed in their relative molar proportions. The dominant composite saccharide of FLBPs was Glc. The minor quantity of Xyl was also detected. DLBPs, by contrast, was made up primarily of Ara and Glc. The proportion of Xyl has increased significantly. This finding was consistent with previous studies [33,34]. The two preparations showed different monosaccharide proportions, especially in Glc and Ara contents. These differences may be associated with differences in raw material state, extraction selectivity, and processing-associated changes. However, monosaccharide composition alone cannot identify the specific contribution of drying or hot-water extraction. Molecular weight (MW) is the important influencing factor of the physicochemical properties and biological activities of polysaccharides [35]. Fig 2A and 2B displays the MW distributions of FLBPs and DLBPs. The distribution profiles suggested that FLBPs dominantly consisted of one macromolecule, which MW was 3712.257 kDa. While DLBPs made up three fractions, which MW were 1068.136 kDa, 122.599 kDa and 33.131 kDa, respectively, indicated that DLBPs was highly dispersive. Polysaccharides is the most important bioactive substance in wolfberry. The HPGPC profiles showed that FLBPs were dominated by a high-MW fraction, whereas DLBPs contained three fractions with lower MWs. This difference may reflect macromolecular degradation [36], extraction selectivity, aggregation/disaggregation [37], or a combination of these processes during conventional dried-material processing.

The structure and type of polysaccharide functional groups can be analyzed by FT-IR [38]. Fig. 2C exhibits the infrared spectra of FLBPs and DLBPs. As shown in Fig 2C, the FTIR spectra of FLBPs and DLBPs showed similar major absorption bands, indicating that both preparations retained typical polysaccharide features. The broad absorption peak at 3288 cm^-1^ was assigned to O-H stretching vibrations involved in intra- and intermolecular hydrogen bonding [39], while the band near 2930 cm^-1^ corresponded to C-H stretching vibrations of sugar residues. The absorption bands at approximately 1580 and 1383 cm^-1^ were associated with carboxylate-related vibrations and possible protein-associated signals [40,41], which was consistent with the presence of uronic acids and proteinaceous components in both polysaccharides. The strong band at 1039 cm^-1^ was attributed to C-O-C and C-O stretching vibrations in glycosidic linkages and pyranose rings. The bands at 818 and 771 cm^-1^ further supported the presence of pyranose-type sugar residues. Although the main FTIR profiles of FLBPs and DLBPs were similar, local differences in band shape and relative intensity were observed, especially in the O–H stretching region and carbohydrate fingerprint region. DLBPs showed additional or more evident bands/shoulders near 1250, 896, and 616 cm ⁻ ¹, whereas FLBPs showed relatively clearer bands near 818 and 771 cm ⁻ ¹. These differences may reflect variations in hydrogen-bonding environment, associated phenolic/proteinaceous components, glycosidic configuration, or aggregation state. Considering the higher phenolic content and broader molecular-weight distribution of DLBPs, the FTIR results provide supportive, but not definitive, evidence for preparation-associated differences in the molecular environment of the two polysaccharide preparations.

The triple-helix conformation of polysaccharides can be assessed by the Congo red assay [42]. As shown in Fig 2D, FLBPs and DLBPs displayed distinct redshifts relative to the Congo red solution within a low alkaline concentration, indicating the presence of Congo red-responsive ordered conformations. The λ_max_ of DLBPs-Congo red remained unchanged within 0.05–0.15 mol/L NaOH solution, suggested that the polysaccharides took an ordered conformation bonded with Congo red [43]. The similar observation did not take on the absorption curve of FLBPs-Congo red. This result suggests that DLBPs had a stronger Congo red-responsive ordered conformation than FLBPs. This difference may be related to their lower-MW fractions, associated phenolics/proteins, or aggregation state [44].

### The morphology analysis of FLBPs and DLBPs

**Fig 3:**
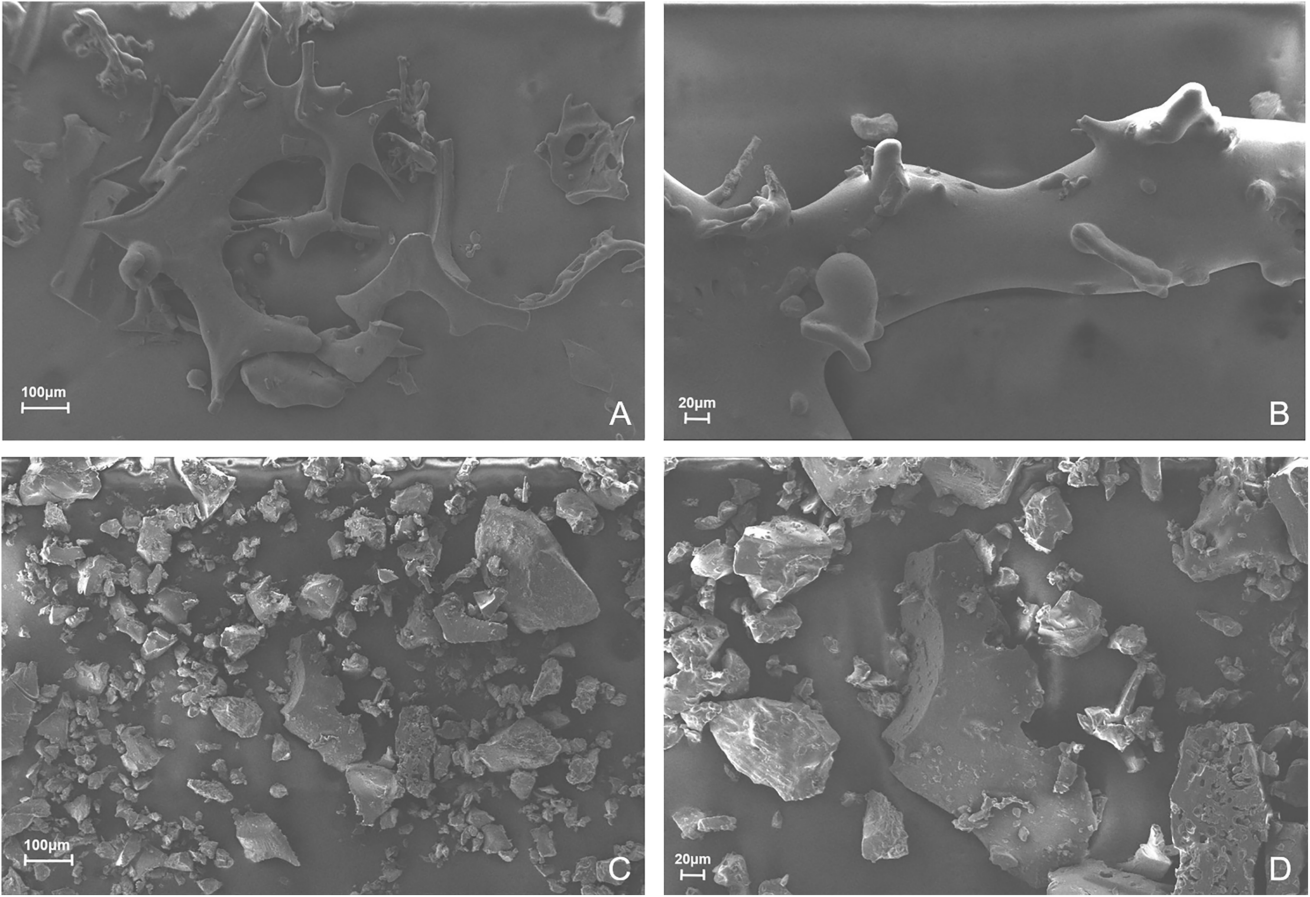
Scanning electron microscope images of FLBPs (A, B) and DLBPs (C, D). Scale bars: A/C = 100 μm, B/D = 20 μm; Images were acquired by the authors.

The scanning electron microscope (SEM) can observe the morphological difference of FLBPs and DLBPs, which showed significant variation in shape. As shown in Fig 3, FLBPs had fluffy and fiber-like structure. In contrast, DLBPs were composed of stone-like particles of varying sizes. Both surfaces were attached with scattered particles. The morphology of DLBPs was consistent with that reported by Ahmadi et al [45]. These morphological differences were consistent with the differences in molecular-weight distribution and solution behavior, suggesting distinct aggregation states between the two preparations.

### The solution behaviors of FLBPs and DLBPs

**Fig 4:**
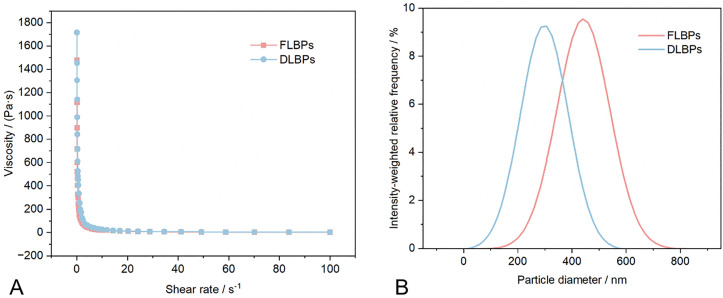
Apparent viscosity, and particle size distribution of FLBPs and DLBPs. Zeta potential values are reported in the main text.

The effects of shear rate on the viscosity of FLBPs and DLBPs (0.2 g/mL) at 25 ℃ are shown in Fig 4. Two crude polysaccharide solutions exhibited shear-thinning behaviors as the present viscosity decreased with increasing of the shear rate from 1 to 100 s^-1^. This phenomenon could be attributed to the disentanglement of molecular chains of solutions [46]. FLBPs’ viscosity was lower than that of DLBPs at low shear rates, which could be attributed to the processing-associated alteration in molecular distribution [47].

Particle size is an important parameter for measuring the size of polysaccharide molecules and can reflect the aggregation state and molecular weight distribution of polysaccharide molecules [48]. While Zeta potential is an indicator for measuring the surface charge of particles. The average particle size of FLBPs (424.29 nm) was larger than that of DLBPs (283.00 nm), which was consistent with MW distribution findings. Generally, polysaccharides with higher MW possessed larger particle size (Fig. 4) [49]. While DLBPs possessed higher homogeneity, evidenced by the smaller PDI (0.21). The smaller average particle size and lower PDI of DLBPs may reflect a more uniform colloidal population, possibly related to the presence of lower-MW fractions and extraction selectivity. The negative zeta potentials of FLBPs and DLBPs suggested that both polysaccharides carried negative surface charges, which was consistent with the presence of uronic acids in the monosaccharide composition. The absolute value of the zeta potential of FLBPs (−22.83 ± 0.78 mV) was higher than that of DLBPs (−21.13 ± 1.81 mV). FLBPs showed a slightly higher absolute zeta potential, suggesting relatively stronger electrostatic stabilization under the tested condition.

Overall, different preparation route may be associated with difference in chemical composition, morphological properties, and solution behaviors of polysaccharides, which may further affect their bioactivities.

### Antioxidant and immunostimulatory activity of FLBPs and DLBPs

#### FLBPs and DLBPs’ cytoprotective activity against H_2_O_2_-induced oxidative stress in HepG2 cells.

**Fig 5:**
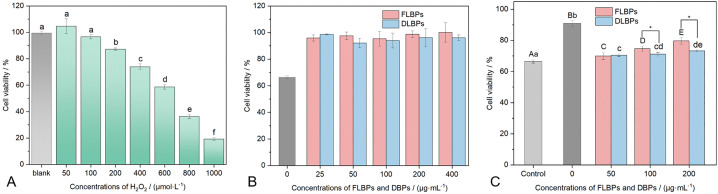
FLBPs and DLBPs’ cytoprotective activity against H_2_O_2_-induced oxidative stress in HepG2 cells. (A) The cell viabilities of HepG2 cell treated by H₂O₂ with different concentrations; (B) The cell viabilities of HepG2 cells treated by FLBPs and DLBPs with different concentrations; (C) The cell viabilities in response to H_2_O_2_-induced oxidative stress in HepG2 cells treated by FLBPs and DLBPs with different concentrations. Values are means of triplicate determination ± standard deviations with different letters are significantly different at *p* < 0.05.

Comparison of chemical evaluation methods, antioxidant assays in living cells is more biologically relevant. H_2_O_2_ can induce oxidative damage in HepG2 cells [50] to access the antioxidant activity of FLBPs and DLBPs. In the study, HepG2 cells were treated with different concentrations of H_2_O_2_ for 12 h (50, 100, 200, 400, 600, 800, 1000 μmol/L), respectively. As shown in Fig 5A, the cell viability displayed a dose-dependent decrease with the increasing concentration of H_2_O_2_. The cell viability significantly decreased when the concentration of H_2_O_2_ reached 200 μmol/L (*p* < 0.05). At 600 μmol/L, H_2_O_2_ reduced the cell viability to 58.8%, so this concentration was used for subsequent experiments.

To evaluate the toxicity of FLBPs and DLBPs, the cells with samples’ solutions were treated with different concentrations (50, 100, 200, 400 μg/mL). The results showed that there was no significant decrease in cell viabilities when polysaccharides solutions increased from 50 to 400 μg/mL, indicating the low cytotoxicity and good biocompatibility of two polysaccharides even at high concentration (Fig 5B).

The cell viability was measured to assess the cytoprotective effects of FLBPs and DLBPs against H_2_O_2_-induce oxidative stress in HepG2 cells. Treatment with tested concentrations (50, 100, 200 μg/mL) of both polysaccharides significantly alleviated H_2_O_2_-induced damage compared with the H_2_O_2_ model group (*p* < 0.05, Fig 5C). At high concentration (100 and 200 μg/mL), FLBPs exhibited better antioxidant activity than those of DLBPs (*p* < 0.05).

Due to the complicated interaction between polysaccharides and cells, it is difficult to conclude the regularity of structure-function relationship based on in vitro cell model. For example, it is still controversial whether MW underlies the antioxidant action of polysaccharides [31]. In the present comparison, FLBPs, which had a higher dominant MW fraction and lower bound phenolic content, showed stronger cytoprotective activity than DLBPs at higher concentrations. This observation suggests an association between macromolecular profile and cellular antioxidant response. However, the individual contributions of MW, phenolic/protein-associated components, and aggregation state cannot be separated in the current design.

#### The effects of FLBPs and DLBPs on the release of inflammatory factors NO and IL-6 from RAW264.7 cells.

While it is well-established that LBPs exhibited immunomodulatory activity [4], how different preparation routes are associated with macrophage-related activity remains insufficiently understood. Thereby, the effects of FLBPs and DLBPs on the release of inflammatory factors NO and IL-6 from RAW264.7 macrophages were investigated.

**Fig 6:**
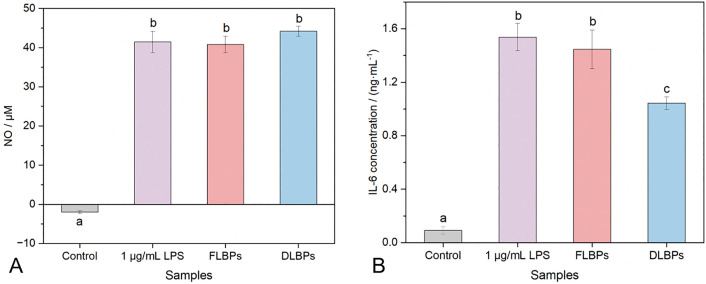
Effects of FLBPs and DLBPs on NO and IL-6 release from RAW264.7 cells. (A) NO release levels from RAW264.7 cells treated with FLBPs and DLBPs; (B) the concentration of IL-6 releases in RAW264.7 cells induced by FLBPs and DLBPs. Values are means of triplicate determination ± standard deviations with different letters are significantly different at *p* < 0.05.

As shown in Fig. 6A, compared to the control group, LPS stimulation significantly induced NO release from RAW264.7 cells, indicating the successful establishment of the cellular inflammation model. Both FLBPs and DLBPs treatment groups showed a strong stimulatory effect on NO release. Their release levels were not significantly different from the LPS group but were significantly higher than those in the control group (*p* < 0.05). The results suggested that two polysaccharides effectively induced the synthesis and release of NO. NO is an important signaling molecule in the immune response of macrophages, and its release level can reflect the activation state of immune cells [51]. These results suggested that both samples could activate macrophage-related responses under the test conditions. The observation of cell morphology further supported this conclusion. Compared to the circular morphology of the control group (S2A Fig), cells in the sample-treated groups showed signs of activation, such as protrusions and spindle shapes (S2B and S2C Fig), consistent with the effect of LPS. Similarly, FLBPs and DLBPs could also induce IL-6 release (Fig 6B), another critical proinflammatory cytokine [52]. The results indicated that two crude polysaccharides could initiate the general activation program of macrophages.

However, FLBPs and DLBPs exhibited differential effects on the release of NO. The results indicated that DLBPs were comparable to LPS in inducing NO release (Fig 6A), while the inductive capacity of FLBPs was significantly weaker (*p* < 0.05). The phenomenon suggested that the different preparation routes may be associated with changes in physicochemical properties, thereby influencing cellular responses.

Taken together, the differences in composition, molecular-weight distribution, Congo red response, particle size, zeta potential, viscosity, and cellular responses indicate that FLBPs and DLBPs represent two polysaccharide preparations with distinct physicochemical and functional profiles. These results support an association between preparation route, macromolecular features, solution behavior, and cellular bioactivity. However, the individual contributions of drying, extraction, molecular-weight distribution, and associated phenolic/proteinaceous components remain to be clarified. Future studies should use matched-process designs, such as fresh and dried materials extracted under comparable conditions and dried samples prepared under controlled drying methods and time gradients, together with purified or reconstituted fractions.

## Conclusion

In this study, a non-thermal extraction strategy was established to obtain polysaccharides from fresh wolfberry. The optimized process involved pulping fresh berries, adjusting the pulp relative density to 1.06, and precipitating polysaccharides with 85% ethanol. The obtained FLBPs were used as a fresh-material reference and compared with DLBPs conventionally extracted from dried wolfberry.

FLBPs and DLBPs showed comparable total carbohydrate and protein contents, but they differed in bound phenolic content, molecular-weight distribution, monosaccharide composition, morphology, conformation-related behavior, solution properties, and cellular bioactivities. DLBPs contained more bound phenolics and displayed a broader molecular weight distribution and stronger macrophage activation, whereas FLBPs showed better cytoprotective activity against H_2_O_2_-induced oxidative stress in HepG2 cells. These results indicate that the two preparation routes generated polysaccharide fractions with distinct physicochemical and functional profiles.

Overall, this study provides a fresh-material reference for evaluating polysaccharides obtained from dried wolfberry by conventional extraction. The observed differences provide useful clues for future studies on how drying, extraction, and associated phenolic or proteinaceous components contribute to the quality attributes of LBPs. Beyond providing a comparative reference, this non-thermal extraction method may offer potential process advantages by reducing repeated drying and heating steps. Further matched-process studies and scale-up evaluation are needed to clarify its practical feasibility.

## Supporting information

S1 Fig Monosaccharide composition analysis. Peaks of mixed monosaccharide standards, FLBPs, and DLBPs.
(DOCX)

S2 Fig The morphology of RAW264.7 cells. Macrophages incubated in DMEM (A), and treated with FLBPs (B) and DLBPs (C) for 24 h, respectively.
(DOCX)

S1 Data Numerical data underlying all figures, tables, and statistical analyses. (XLSX)
