# Supplementary material for: Comparison of non-thermally extracted polysaccharides from fresh wolfberry with conventionally extracted polysaccharides from dried wolfberry
Source: PLoS One. 2026 Aug 11;21(8):e0355472. doi: 10.1371/journal.pone.0355472 (PMC13460608; doi:10.1371/journal.pone.0355472)
Supplement: Supplementary file 1 [file pone.0355472.s001.docx]

**Supporting information**

**S1 Fig. Monosaccharide composition analysis.** Peaks of mixed monosaccharide standards, FLBPs, and DLBPs.
