# Supplementary material for: Comparison of non-thermally extracted polysaccharides from fresh wolfberry with conventionally extracted polysaccharides from dried wolfberry
Source: PLoS One. 2026 Aug 11;21(8):e0355472. doi: 10.1371/journal.pone.0355472 (PMC13460608; doi:10.1371/journal.pone.0355472)
Supplement: Supplementary file 2 [file pone.0355472.s002.docx]

**Supporting information**

**S2 Fig. The morphology of RAW264.7 cells.** Macrophages incubated in DMEM (A), and treated with FLBPs (B) and DLBPs (C) for 24 h, respectively.
